# A heterozygous variant in the human cardiac miR-133 gene, *MIR133A2*, alters miRNA duplex processing and strand abundance

**DOI:** 10.1186/1471-2156-14-18

**Published:** 2013-03-06

**Authors:** Monique Ohanian, David T Humphreys, Elizabeth Anderson, Thomas Preiss, Diane Fatkin

**Affiliations:** 1 Molecular Cardiology Division, Victor Chang Cardiac Research Institute, Darlinghurst, New South Wales, Australia; 2 Molecular Genetics Division, Victor Chang Cardiac Research Institute, Darlinghurst, New South Wales, Australia; 3 Faculty of Medicine, University of New South Wales, Kensington, New South Wales, Australia; 4 Capital Cardiac Centre, Garran, Australian Capital Territory, Australia; 5 Genome Biology Department, The John Curtin School of Medical Research, The Australian National University, Canberra, Australian Capital Territory, Australia; 6 Cardiology Department, St Vincent’s Hospital, Darlinghurst, New South Wales, Australia

**Keywords:** MicroRNA, isomiR, Genetics, Atrial fibrillation

## Abstract

**Background:**

MicroRNAs (miRNAs) are small non-coding RNAs that post-transcriptionally regulate gene expression. Sequential cleavage of miRNA precursors results in a ~22 nucleotide duplex of which one strand, the mature miRNA, is typically loaded into the RNA-induced silencing complex (RISC) while the passenger strand is degraded. Very little is known about how genetic variation might affect miRNA biogenesis and function.

**Results:**

We re-sequenced the *MIR1-1, MIR1-2, MIR133A1*, *MIR133A2*, and *MIR133B* genes, that encode the cardiac-enriched miRNAs, miR-1 and miR-133, in 120 individuals with familial atrial fibrillation and identified 10 variants, including a novel 79T > C *MIR133A2* substitution. This variant lies within the duplex at the 3^′^ end of the mature strand, miR-133a-3p, and is predicted to prevent base-pairing and weaken thermostability at this site, favoring incorporation of the passenger strand, miR-133a-5p, into RISC. Genomic DNA fragments containing miR-133a-2 precursor sequences with 79T and 79C alleles were transfected into HeLa cells. On Northern blotting the 79T allele showed strong expression of miR-133a-3p with weak expression of miR-133a-5p. In contrast, the 79C allele had no effect on miR-133a-3p but there was a significant increase (mean 3.6-fold) in miR-133a-5p levels. Deep sequencing of small RNA libraries prepared from normal human and murine atria confirmed that nearly all the mature miR-133a was comprised of miR-133a-3p and that levels of miR-133a-5p were very low. A number of isomiRs with variations at 5^′^ and 3^′^ ends were identified for both miR-133a-3p and miR-133a-5p, with 2 predominant miR-133a-3p isomiRs and one predominant miR-133a-5p isomiR. Bioinformatics analyses indicate that the major miR-133a-3p and 5p isomiRs have numerous predicted target mRNAs, only a few of which are in common.

**Conclusions:**

Multiple miR-133a isomiRs with potential different mRNA target profiles are present in the atrium in humans and mice. We identified a human 79T > C *MIR133A2* variant that alters miRNA processing and results in accumulation of the miR-133a-5p strand that is usually degraded.

## Background

MicroRNAs (miRNAs) are small non-coding RNAs that regulate gene expression. They act by binding to the 3^′^untranslated regions of target mRNAs and typically reduce mRNA levels by inhibiting translation or stimulating mRNA decay [1]. MiRBase v18 [2] lists 2148 human miRNAs, each of which is expected to have multiple mRNA targets. Consequently, miRNAs can orchestrate suites of changes in gene expression and have been implicated in diverse normal physiological processes and disease states [3].

Most miRNAs are transcribed from their own genes by RNA polymerase II. A minority of miRNAs are located within introns of other genes and are co-transcribed with the host gene. In the canonical miRNA biosynthesis pathway, primary miRNA (pri-miRNA) hairpins are cleaved by the nuclear RNAase III-type endonuclease Drosha to yield a ~70 nucleotide (nt) precursor miRNA (pre-miRNA) that is then exported to the cytoplasm. Further cleavage of this stem-loop structure y Dicer generates a ~ 22 nt mature miRNA duplex [4]. Typically, the dominant (mature) strand of the duplex is incorporated into an RNA-induced silencing complex (RISC) that binds to the target mRNA by sequence-specific interactions between the “seed” region (nt 2–8) at the 5^′^ end of the miRNA and the cognate mRNA binding site. The remaining (passenger) strand of the duplex is thought to be non-functional and degraded. Recent deep sequencing data have revealed surprising additional complexity of miRNA processing and have found that multiple miRNA isoforms (isomiRs) of varying sequence composition and length may be present [5-8].

The muscle-enriched miRNAs, miR-1 and miR-133, are amongst the most abundant of the miRNAs present in the normal heart [9,10]. Two genes, *MIR1-1* and *MIR1-2*, encode miR-1-1 and miR-1-2, while three genes, *MIR133A1*, *MIR133A2*, and *MIR133B*, encode miR-133a-1, miR-133a-2, and miR-133b, respectively. MiR-133a-1 and miR-133a-2 have identical mature sequences, with miR-133b differing only by a single nt at the 3^′^ end. In humans, *MIR1-1* and *MIR133A2* are located on chromosome 20 while *MIR1-2* and *MIR133A1* are located on chromosome 18. These 2 pairs of genes are co-regulated and expressed as bicistronic transcripts. *MIR133B* is on chromosome 6 and is paired with another muscle-specific miRNA gene, *MIR206*. Verified mRNA targets of miR-1 and miR-133 include those encoding proteins that are involved in cardiac development, ion channel function, hypertrophy, and fibrosis [11-16].

Atrial fibrillation (AF) is the most common cardiac arrhythmia and a frequent complication of diverse cardiac and systemic disorders. Altered levels of miR-1 and miR-133 have been observed in atrial tissue samples from patients with AF in several studies [17-19]. MiRNA-induced gene expression changes in the atrium could either have a primary role in initiation of AF or contribute to electrical and structural remodelling that perpetuates AF. In this study, we hypothesized that genetic variation could alter the functional effects of miR-1 and miR-133 and contribute to AF pathogenesis. To test this hypothesis, we performed genetic screening of the *MIR1-1, MIR1-2, MIR133A1*, *MIR133A2*, and *MIR133B* genes in a cohort of probands with suspected familial AF. A number of variants were identified, including a novel *MIR133A2* variant that was functionally characterized and found to alter miR-133a duplex processing. To assess the potential effects of this variant, we first needed to catalogue the abundance and diversity of miR-133a isomiRs in the normal heart. Deep sequencing of human and murine atrial tissue was performed and revealed an unexpected diversity of miR-133a isomiRs, with nearly all the miR-133a tags comprised of the 2 major miR-133a-3p isomiRs and <1% comprised of miR-133a-5p species. Our data suggest that the *MIR133A2* variant increases the relative abundance of miR-133a-5p.

## Results

### MiR-1 and miR-133 sequence variants

**Figure 1:**
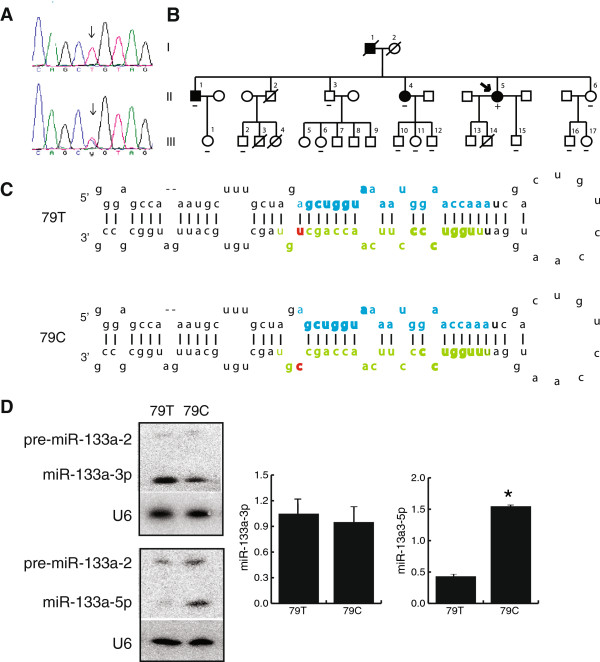
**Characteristics of the 79T > C *MIR133A2 *variant.** (**A**) Sequence electropherograms with arrows indicating normal 79T allele (top panel) and variant 79C allele (lower panel). (**B**) Family KB pedigree. Phenotypes are indicated as: AF (solid symbols), no AF (open symbols). The presence (+) or absence (−) of the 79T > C *MIR133A2* variant is shown and the family proband is indicated by an arrow. (**C**) Stem-loop region of precursor miR-133a-2 transcript depicting the most abundant human isomiRs for miR-133a-3p (green) and miR-133a-5p (blue) with seed regions indicated (bold). The 79T > C variant (red) is located at the 3′end of miR-133a-3p directly adjacent to the Drosha cleavage site. (**D)** Northern blot probed with miR-133a-3p (top panel) and miR-133a-5p (lower panel) showing expression levels of 79T and 79C alleles. Mean data for triplicates of each sample are indicated on bar graphs with 79C values expressed relative to 79T values. *P <0.05 when compared to 79T.

The 5 loci encoding miR-1 and miR-133 precursor transcripts were re-sequenced in 120 probands with a family history of AF. Ten variants were identified, 2 of which were novel (Table 1). For the 8 known variants, the minor allele frequencies in the AF group were similar to those reported in the public databases, dbSNP, NHLBI Exome Variant Server and 1000 Genomes. Three *MIR133A2* variants, -102G > A, -82G > A, and -19G > A, were in linkage disequilibrium as reported previously [20]. A *MIR133A2* haplotype that included -102G > A, -82G > A, -19G > A, and +47T > C was present on one allele in 18 AF probands (15%) and 37 in-house controls (15%), and on both alleles in 8 AF probands (7%) and 12 in-house controls (5%). Of the 2 novel variants, only one was located within a miRNA stem-loop sequence. This variant, a 79T > C substitution in the *MIR133A2* gene (Figure 1A), was found in a 67-year old female (II-5, Family KB, Figure 1B) who had paroxysmal episodes of AF, hypertension and mitral valve disease. The two other living family members with AF, II-1 and II-4, were genotype-negative. The 79T > C variant was not seen in clinically unaffected relatives, in our 250 healthy control subjects, or in the human genome sequence databases.

**Table 1:** MiR-1 and miR-133 gene variants identified in familial AF probands

**Gene**	**Chromosome**	**Nucleotide change**^**a**^	**Major/Minor allele**	**Minor allele frequency**^**b**^				**dbSNP**^**c**^
				**AF**	**Controls**	**ESP**	**1000G**	
*MIR1-1*	20	−43C > T	C/T	0.004	NA	-	-	Novel
*MIR1-2*	18	+15A > G	A/G	0.017	NA	0.020	0.018	rs9989532
		+74T > C	T/C	0.046	NA	0.087	0.071	rs78641532
*MIR133A2*	20	−102G > A	G/A	0.142	0.122	-	0.207	rs45547937
		−82G > A	G/A	0.142	0.122	-	0.206	rs13040566
		−19G > A	G/A	0.142	0.122	0.229	0.207	rs13040413
		79T > C	T/C	0.004	0	-	-	Novel
		+47T > C	T/C	0.400	0.340	0.388	0.387	rs6062251
		+69G > A	G/A	0.004	0.010	0.006	0.009	rs149629841
*MIR133B*	6	−25delA	A/-	0.083	NA	0.075	0.074	rs142410335

^a^ Location of nucleotide changes is shown as (−) downstream or (+) upstream of the miRNA stem-loop sequence (as annotated by miRBase).

^b^*AF*, atrial fibrillation (n = 120, this study cohort); Controls, healthy volunteer subjects (n = 250, this study cohort); *ESP*, NHLBI Exome Sequencing Project (European American subgroup data); 1000G, 1000Genomes Project (European subgroup data). *NA*, data not available (not tested).

^c^ dbSNP, Database of Single Nucleotide Polymorphisms [ http://www.ncbi.nlm.nih.gov/projects/SNP/].

### 79T > C MIR133A2 variant increases levels of miR-133a-5p

The 79T > C *MIR133A2* variant is positioned directly adjacent to the Drosha cleavage site in the stem-loop structure at the 3^′^ end of miR-133a-3p (Figure 1C). In the absence of sequence data for human miR-133a-5p in miRBase, this location was initially deduced from 3p dominant processed sequence listed for mouse. We predicted that the mismatch introduced by the 79T > C substitution would alter secondary structure and thus precursor processing and/or subsequent strand selection. To test these predictions, we prepared two constructs that replicated the 79T and 79C genotypes of mature miR-133a, transfected these into the HeLa cell line that does not detectably express endogenous miR-133a and performed Northern blotting. For these studies, the miR-133a-2 haplotype (−19G > A, +47T > C), which was present in the proband, II-5, and her affected sister, II-4, was used as the background sequence. The 79T allele had strong expression of miR-133a-3p with low amounts of miR-133a-5p. When compared with 79T, the 79C allele showed no significant change in miR-133a-3p (*P* = 0.81) but there was a mean 3.6-fold increase in levels of miR-133a-5p (*P* = 0.003, Figure 1D).

### MiR-133a populations in the normal atrium

**Figure 2:**
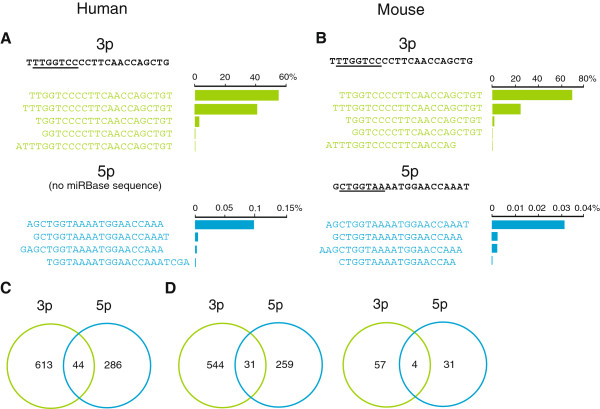
**Abundance of miR-133a 5**^′ ^**isomiRs in atrial tissue.** MiR-133a-3p (green) and miR-133a-5p (blue) isomiRs were evaluated by deep sequencing of human (**A**) and murine (**B**) atrial tissues. 5^′^ isomiRs affect the seed sequences and are considered functionally more important than 3^′^ isomiRs. The most abundant 5^′^ isomiRs, with the most frequently-occurring 3^′^variant for each of these, are shown. MirBase-annotated miR-133a-3p and 5p sequences are shown in black with ‘seed’ regions (nt 2–8) underlined. Note that there is no miRBase annotation for human miR-133a-5p. (**C)** Venn diagram showing numbers of human target mRNAs predicted by TargetScan with the seed regions of the 2 most abundant human miR-133a-3p isomiRs and the single abundant miR-133a-5p isomiR used as inputs. (**D**) (Left panel) Murine target mRNAs predicted by TargetScan with seed regions of the 2 abundant murine miR-133a-3p isomiRs and single abundant miR-133a-5p isomiRs as inputs. (Right panel) Venn diagram showing numbers of putative miR-133a-3p and miR-133a-5p target mRNAs determined by cross-referencing predicted murine targets with genes demonstrated to be differentially regulated in the hearts of miR-133a knockout mice [22].

To determine the diversity and abundance of miR-133a-3p and 5p processed species that are normally present in the atrium, small RNA libraries were prepared from human and murine heart tissue samples and were subjected to deep sequencing. A comprehensive list of the sequences and expression levels of all the cardiac miRNA isomiRs identified is provided in the (Additional file 1: Table S1 and Additional file 2: Table S2). In human and murine atrial tissues, miR-133 was the most highly expressed miRNA, comprising approximately 20% of all miRNA sequences. Analysis of sequencing tags that map to a miR-133a locus showed an extensive range of 5^′^ and 3^′^ isomiRs for miR-133a-3p and miR-133a-5p in both human and mouse (Additional file 3: Table S3 and Additional file 4: Table S4). There were two predominant isomiRs processed from the miR-133a 3p arm in both the human and murine atria (Figures 2A and 2B). The sequences for the miR-133a high-abundance isomiRs were identical in the two species. Together, the abundance of these tags represented >99% of all tags derived from the miR-133a hairpin. Interestingly, the more abundant sequence in both human and murine samples was not the sequence annotated as the mature form in miRBase, but rather the +1 isomiR. Less than 1% of all sequences that mapped to the miR-133a hairpin aligned to the 5p arm. There was a single predominant miR-133a-5p isomiR in human atrium, which started one nt upstream from the murine miRBase entry. There was also only one major murine miR-133a-5p isomiR that had an identical 5^′^ sequence to the major human miR-133a-5p isomiR.

### MiR-133a mRNA target profiles

**Figure 3:**
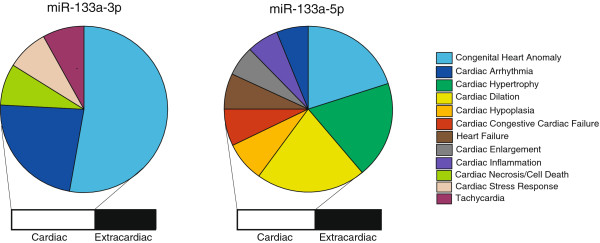
**Functional analysis of human miR-133a-3p and miR-133a-5p targets.** Significant associations were found in the Ingenuity Knowledge Base between predicted human target mRNAs of miR-133a-3p and miR-133a-5p and a range of cardiac and extra-cardiac biological functions and/or disease states. Parameters that achieved statistical significance (*P* < 0.05) are shown for miR-133a-3p (left) and miR-133a-5p (right), with the relative proportions determined on the basis of the negative logarithm of the *P* values.

We hypothesized that increases in miR-133a-5p associated with the 79T > C *MIR133A2* variant could result in selective down-regulation of a distinctive set of target mRNAs. To explore what these cardiac genes might be, we used the seed sequences of the two most abundant human miR-133a-3p isomiRs and the single abundant miR-133a-5p isomiR identified by deep sequencing to look for predicted human mRNA binding sites in TargetScan. For the two abundant human miR-133a-3p isomiRs, the seed sequences TGGTCCC and TTGGTCC had 402 and 502 predicted targets, respectively, of which 247 were in common. Only 26 (6%) of the total 402 TGGTCCC human miR-133a-3p isomiR targets overlapped with the predicted targets for the abundant human miR-133a-5p, and similarly, 30 (6%) of the total 502 TTGGTCC isomiR targets overlapped with those predicted for human miR-133-5p. Overall, only 44 (7%) of the total 657 human miR-133a-3p predicted targets from both abundant isomiRs overlapped with the targets predicted for miR-133a-5p (Figure 2C). To compare murine miR-133a-3p and miR-133a-5p targets, we used the two predominant 3p and single predominant 5p murine isomiR seed regions to search for predicted mRNA binding sites in TargetScan. Similar to the human data, only a minority of murine miR-133a-3p targets (31 [5%]) overlapped with 5p targets (Figure 2D). When all TargetScan-predicted targets were considered, there was substantial concordance across species with 514 (78%) of human miR-133a-3p targets and 259 (78%) of human miR-133a-5p targets shared by mouse. We then cross-referenced all the predicted murine targets to a set of mRNAs identified by Liu and colleagues [21] that showed altered expression levels in miR-133a knockout mice. This produced a list of 61 predicted targets for miR-133a-3p and 35 predicted targets for miR-133a-5p, only 4 of which were in common (Figure 2D). We also compared our TargetScan outputs to a list of 1640 miR-133a targets identified by Matkovich and colleagues in mouse heart using RISC-seq [16]. When the TargetScan and RISC-seq data were compared, there were 168 miR-133a-3p targets when considering both major isomiRs, and 75 miR-133a-5p targets, only 6 of which were common. Matkovich and colleagues reduced their list of 1640 targets to 209 by analyzing transgenic mice that overexpress miR-133a [16]. A comparison of TargetScan targets with this refined list identified 27 miR-133a-3p targets when considering both major isomiRS and 4 miR-133a-5p targets, with no targets in common. These findings collectively suggest that miR-133a isomiRs have distinctive target spectra. In the Ingenuity Knowledge Base, the TargetScan-predicted human miR-133a-3p and 5p target mRNAs are associated with a range of cardiac and extra-cardiac biological functions (Figure 3).

## Discussion

There are two major outcomes of this study. First, we have characterized atrial miR-133a species and find an extraordinary diversity of 5^′^ and 3^′^ miR-133a-3p and miR-133a-5p isomiRs, with two major 3p isomiRs and one major 5p isomiR in both human and mouse. These isomiRs have minimally-overlapping suites of predicted mRNA targets and functions, which suggests that they have distinct biological roles. Second, we report a novel *MIR133A2* variant that alters strand abundance during miRNA processing and results in accumulation of miR-133a-5p. This variant is one of only a very few functional miRNA gene variants reported to date.

Despite the enormous interest in miRNAs as master regulators of gene expression, the role of genetic variation is incompletely understood. Single nucleotide polymorphisms (SNPs) in predicted miRNA binding sites on target mRNAs occur frequently and some of these have been associated with phenotypic traits [22-25]. In contrast, SNPs in the miRNA genes themselves are far less common. Several SNPs in pri-miRNA and pre-miRNA have been functional evaluated and shown to result in defective processing and reduced levels of mature miRNA. SNPs are rarely seen (<1%) in the seed regions that are crucially required for target recognition [22]. These observations have provided an argument for strong selective constraint and suggest that seed variants would have substantial effects. The discovery of point mutations in the seed region of miR-96 in two families with nonsyndromic progressive hearing loss provided the first example of human Mendelian disease associated with a miRNA gene variant [26]. Functional SNPs can occur in mature miRNAs outside the seed region, as recently demonstrated by the finding of a rare variant at nt 17 of miR-499 [27]. Evaluation of the effects of this variant in transgenic mice showed that it protected against the cardiomyopathy that developed with overexpression of the wildtype form of miR-499. Here we describe a *MIR133A2* variant located in the mature miRNA outside the seed region that changes the way that the miRNA duplex is processed.

MiRNA processing is conventionally assumed to yield only one functional mature miRNA with the passenger strand being degraded and hence, functionally irrelevant. It has recently been appreciated however, that passenger forms are not always selected for degradation, rather, that both 3p and 5p strands of the miRNA duplex may be simultaneously present and that their ratio can be regulated in a tissue- or development-specific manner, suggesting that both strands have inherent functional activity [28]. Here we find that both (mature) miR-133a-3p and (passenger) miR-133a-5p are present in the atrium in humans and mice, with miR-133a-5p normally representing <1% of all miR-133a species. The main effect of the 79T > C *MIR133A2* variant is to alter the relative ratio of miR-133a-3p and 5p strands.

Current models posit that the strand of the miRNA duplex with weaker base-pairing at the 5^′^ end is preferentially incorporated into RISC [4]. If the conventional murine miR-133a-5p sequence as annotated by miRBase (v18) represented the predominant isomiR in the normal human heart, then the nt corresponding to position 79 would lie just outside the base-paired region of the processed miRNA duplex. However, our deep sequencing data clearly show that the −1 isomiR is the most abundant miR-133a-5p species in the human atrium. Consequently, the 79T > C variant lies within the duplex and would directly prevent base-pairing and weaken thermostability at this site, favoring incorporation of miR-133a-5p into RISC. Additionally the lack of base pairing at position 79C is likely to enlarge the bulge in the stem-loop structure, a feature known to influence positional cleavage by Dicer and Drosha and result in increased variability of 5^′^ isomiRs [8,29]. The canonical miRNA biosynthesis pathway predicts 3p and 5p miRNAs of 22 nt in length, but deep sequencing studies have revealed a surprising extent of isomiR diversity in most tissues and species [5-8]. These isomiRs may include 5^′^ or 3^′^ cleavage variations, and non-templated additions or trimming at the 3^′^ end. It has been found that the relative abundance and types of isomiRs in specific tissues can vary over time and with disease states, suggesting that they have distinctive biological functions [5,8,30].

It is conventionally assumed that the seed regions are the major determinants of target specificity, and thus it is plausible that 5^′^ isomiR variation can affect mRNA target selection. For example, our group has recently demonstrated that the two most abundant miR-133a isomiRs in murine atrial HL-1 cells have different targeting properties [8]. Given that each isomiR has hundreds of mRNA targets, dynamic fluxes in isomiR characteristics may give rise to an unexpected complexity of miRNA:mRNA interactions and provide a mechanism for intricate regulation of gene expression in specific tissues. To assess the potential impact of the 79T > C *MIR133A2* variant, we searched for differences in miR-133a-3p and miR-133a-5p predicted mRNA targets using the respective human and murine seed regions and a set of genes known to be differentially regulated in miR-133a knockout mice [21]. These results showed that only a minority of predicted miR-133a targets were shared, and that most were unique to either 3p or 5p forms. With the 79T > C *MIR133A2* variant, no changes in miR-133a-3p target gene expression would be expected. In contrast, the relative increase in miR-133a-5p could have a relatively greater impact and give rise to selective repression of the 5p suite of targets. It is notable that at least 25% of the lowest-abundance miR-133a-5p targets include mRNAs involved in regulation of transcription, signaling and membrane transport. Further studies are required to determine whether changes in miR-133a-5p directly alter levels of these critical molecules and have biologically-significant functional effects.

The relationship, if any, between the 79T > C *MIR133A2* variant and AF in the Family KB proband is questionable. Although DNA samples were unavailable from the deceased parents, it seems most likely that the 79 T > C substitution is a *de novo* sequence change which was uniquely present in one family member. A causal gene mutation that can account for the familial pattern of AF in this kindred has yet to be identified. The presence of the *MIR133A2* variant in the Family KB proband could foreseeably have a modifying effect by altering gene expression profiles in the atrium. A number of factors in addition to genetic variation need to be considered in this individual, including the impact of AF *per se* and of co-existent risk factors for AF. Altered expression of miR-133 itself has been observed in cardiac tissues from patients with AF [18,19], and conditions that predispose to AF, such as atrial dilation, ventricular hypertrophy, and myocardial ischemia [12,31]. It is notable that individual II-5 had both hypertension and valvular heart disease which independently increase AF risk. A limitation of this study is the lack of atrial tissue samples to document gene expression patterns in 79T and 79C family members. The collective effects of the multiple variables present in individual II-5 on atrial gene expression and the electrical and structural properties of the atrial wall are difficult to predict and could promote or protect against AF.

## Conclusions

MiRNA duplex processing generates a dominant strand that is incorporated into RISC and a passenger strand that is usually degraded. In the normal human atrium, almost all the miR-133a is comprised of miR-133a-3p with negligible amounts of miR-133a-5p. Although multiple 5^′^ and 3^′^ isomiRs are present, there are only 2 major miR-133a-3p isomiRs and one major miR-133a-5p isomiR. These isomiRs have distinctive suites of predicted gene targets suggesting independent biological roles. Very few genetic variants in mature miRNA sequences have been reported and functionally characterized. We have identified a missense *MIR133A2* variant that alters miR-133a duplex processing and strand abundance with accumulation of miR-133a-5p in HeLa cells. Further studies to confirm these findings and to document mRNA expression profiles in patient heart tissues are indicated. Despite the importance of miR-133a in atrial biology, miR-133a genetic variants are not a common cause of familial AF.

## Methods

### Genetics study subjects

One hundred and twenty subjects with suspected familial AF were identified from St Vincent’s Hospital and by physician referral. Family probands and their relatives were evaluated by medical history and physical examination, 12-lead echocardiography and transthoracic echocardiography. A positive family history was defined as the presence of documented AF in 2 or more first-degree family members. Two hundred and fifty healthy individuals with no history of cardiovascular disease comprised a control group. All families and control subjects were of Western European background. Genomic DNA was isolated from whole blood samples using the standard salting-out technique. Informed, written consent was obtained from all participants and protocols were approved by the St Vincent’s Hospital campus human and animal research ethics committees.

### Genetic analysis

*MIR1-1, MIR1-2, MIR133A1*, *MIR133A2*, and *MIR133B* were re-sequenced in DNA samples from family probands. Primer sequences were designed to amplify regions spanning at least 120 bp upstream and downstream of the stem-loop sequences (as annotated by miRBase) of hsa-miR-1-1, hsa-miR-1-2, hsa-miR-133a-1, hsa-miR-133a-2, hsa-miR-133b (miRBase accession numbers MI0000651, MI0000437, MI0000450, MI0000451, MI0000822, respectively). Polymerase chain reaction (PCR) was carried out on genomic DNA using FastStart Taq DNA Polymerase (Roche, Indianapolis, IN, USA) on a Dyad Peltier Thermal Cycler (Bio-Rad Laboratories, Hercules, CA, USA) with the primer pairs shown in Table 2, designed using the programs *Primer3Plus* and *Amplify* version 3.1.4 (University of Wisconsin, Madison, WI, USA). Cycling conditions for *MIR1-1*, *MIR133A2* and *MIR133B* are as follows: 94°C (3 min), then 35 cycles of 94°C (20 s), 55°C (30 s), 72°C (60 s) and finally 72°C (8 min). *MIR1-2* and *MIR133A1* were amplified using the “Slowdown PCR” method. Amplicons were purified with ExoSAP-IT enzyme (GE Healthcare, Chalfont St Giles, Buckinghamshire, UK), sequenced using Big Dye Terminator (version 3.1, Applied Biosystems, Foster City, CA, USA) and analyzed on an ABI PRISM 3700 DNA Analyzer (The Ramaciotti Centre for Gene Function Analysis, UNSW, Sydney, New South Wales, Australia). Viewing and analysis of the data was carried out using the DNASTAR software package (Madison, WI, USA). DNA sequence variants were validated by re-sequencing independent PCR-generated amplicons from probands in both directions. Sequence variants identified in family probands were subsequently evaluated in affected and unaffected family members and in the cohort of healthy control subjects.

**Table 2:** Primer sequences used for PCR amplification and sequencing

**Primer name**	**Primer sequence**	**Amplicon size (bp)**	**Sequencing primer**
mir-1-1F	5^′^- gagatggattcagggatgga -3^′^	494	mir-1-1R
mir-1-1R	5^′^- acctgctgacacaggcaaag -3^′^		
mir-1-2F	5^′^- ggaaccattaatgccatgct -3^′^	467	mir-1-2F
mir-1-2R	5^′^- tgaaatctacttcactggatcttctt -3^′^		
mir-133a-1F	5^′^- tttaaaccattaagcgcagga -3^′^	455	mir-133a-1F
mir-133a-1R	5^′^- ttgaaatccttaagtcatccataca -3^′^		
mir-133a-2F	5^′^- ctgcagagcttgagggaaac -3^′^	466	mir-133a-2R
mir-133a-2R	5^′^- caaggaggaacaagcaggag -3^′^		
mir-133bF	5^′^- agtcatgcaacatgaaatacaaa -3^′^	500	mir-133bR
mir-133bR	5^′^- gagtgcaaaggcacagaaca -3^′^		

### Plasmid constructs and cell transfections

A 272bp fragment containing the precursor sequence of mature miR-133a-2 with 79T and 79C alleles was amplified from genomic DNA using gene-specific primers containing BamHI and HindIII restriction enzyme sites (underlined): forward: 5^′^- ggggcggccgcggatccatctccatcgggactgc -3^′^, reverse: 5^′^- gggaagcttggcactcagggcttcactta -3^′^. Amplification was carried out using FastStart Taq DNA Polymerase with the same cycling condictions as described for *MIR1-1*, *MIR133A2* and *MIR133B* above. PCR amplicons were purified using the QIAquick Gel Extraction Kit (Qiagen, Hildem, Germany) and cloned into the pGEM®-T Easy Vector system (Promega, Fitchburg, WI, USA) according to manufacturer’s instructions. Clones were digested with BamHI and HindIII and ligated to BamHI- and HindIII-digested p*Silencer*^TM^ puro 4.1-CMV vector (Applied Biosystems). All plasmid constructs were confirmed by sequencing.

HeLa cells (ATCC, Manassas, VA, USA) were maintained in Dulbecco’s modified eagle medium (DMEM) with 5% fetal calf serum, supplemented with glutamine and penicillin-streptomycin. Cells were seeded in a 6-well plate 8 hours before transfection. Transfections were performed in duplicate at ~70% confluency by adding 1.5mL of DMEM and 1mL of transfection solution per well. The 1mL transfection solution contained Opti-MEM®, 150ng of 79T or 79C constructs and 5uL of Lipofectamine 2000 reagent (Invitrogen, Carlsbad, CA, USA), which was incubated for 20 minutes at room temperature prior to transfection.

### RNA analysis

HeLa cells (ATCC) were harvested after 24 hours and total RNA was extracted using TRIzol® (Invitrogen). Total RNA (5ug) was separated on 5% stacking/12% resolving polyacrylamide gels, transferred electrophoretically to Gene Screen Plus® Hybridization Transfer Membranes (Perkin Elmer, Waltham, MA, USA). Separated RNA was fixed on the membranes by ~20 sec UV crosslinking on a UV Stratalinker 2400 (Stratagene, Santa Clara, Ca, USA). Northern blotting was performed as described [32]. DNA oligonucleotide probes (5^′^- tacagctggttgaaggggaccaaa -3^′^, 5^′^- gatttggttccattttaccagct -3^′^, 5^′^- tgtgctgccgaagcaagcac -3^′^) complementary to mature miR-133a (miR-133a-3p), passenger miR-133a (miR-133a-5p) and U6 sequences, respectively, were end-labeled with ^32^P using T4 Polynucleotide Kinase (New England Biolabs, Ipswich, MA, USA) and purified by Microspin G-25 columns (GE Healthcare) according to manufacturer’s instructions. A single probe was incubated with the membrane in hybridization buffer (250mM NaPO_4_, 7% SDS, pH 7) at 42°C overnight. After hybridization, membranes were washed 3 times with 2xSSC (1.753% NaCl, 0.882% Na_3_C_6_H_5_O_7_) at 42°C, and exposed overnight. Phosphorimaging was performed on a Fuji imager (FLA-5100, Fujifilm, Minato-ku, Tokyo, Japan). Hybridization signals were quantified using Multi Gauge v2.3 software (Fujifilm, Minato-ku, Tokyo, Japan) and the values measured for each probe from each blot were scaled to the average signal. The scaled miR-133a values were then normalized to the scaled U6 values to adjust for any loading bias. Statistical analyses were performed using the Mann–Whitney *U* test with a *P* value <0.05 deemed significant.

### Deep sequencing

For deep sequencing of atrial myocardium, total RNA was extracted using the miRNeasy® Kit (QIAGEN, Hildem, Germany) from right atrial appendage samples obtained from two males aged 62 and 64 years with no history of AF who underwent coronary artery bypass graft surgery. Atrial tissue was also obtained from the hearts of 3 wild-type mice aged 4–6 weeks. Small RNA libraries were created using NEBNext® small RNA library preparation kit (New England Biolabs, Ipswich, MA, USA) and sequenced on a 5500 SOLiD^TM^ platform (Applied Biosystems). Sequencing data were mapped using Lifescope software (Applied Biosystems). Processed miRNA counts were initially collated from all tags that aligned within 3 nt of the miRBase defined 5′ starting position. Tags that mapped to miRBAse v18 can be accessed via a “miRDSPRing” document (D. Humphreys, manuscript in preparation) and are available in the Supporting Information (Tables S1 and S2).

### MiRNA target predictions

Gene targets for human and murine miR133a were predicted using TargetScan Custom (v5.2) [33] with the “seeds” (nt 2–8) of the most abundant miR-133a-3p and miR-133a-5p isomiRs that were identified via deep sequencing used as inputs. The newer version of TargetsScan (v6.2) and a range of other commonly-used publically-available target prediction programs (DIANA-microT, Microinspector, microRNA.org, MirTarget2, PicTar, PITA, RNA22, RNAhybrid) were unable to be utilized because human and/or murine miR-133a-5p sequences were unable to be inputted and/or analyzed. Predicted gene targets of murine miR-133a were matched against mRNAs that in which expression levels were altered by at least 1.5 fold in microarray data from miR-133a knockout mice [21]. The TargetScan outputs for human miR-133a-3p and miR-133a-5p were imported into Ingenuity Pathway Analysis software (Ingenuity® Systems, http://www.ingenuity.com) for identification of biological pathways in the heart that might be differentially regulated (Additional file 5: Table S5).

## Abbreviations

AF: Atrial fibrillation; DMEM: Dulbecco’s modified eagle medium; miRNA: microRNA; nt: Nucleotide; PCR: Polymerase chain reaction; pre-miRNA: Precursor miRNA; pri-miRNA: Primary miRNA; RISC: RNA-induced silencing complex

## Competing interests

The authors declare that they have no competing interests.

## Authors’ contributions

MO performed the genetics studies, plasmid construct preparations, cell transfections, RNA analyses, miRNA target predictions and drafted parts of the manuscript. DTH carried out RNA analyses and performed deep sequencing experiments and analysis. EA performed clinical evaluation of family members. TP participated in the design of the study and provided intellectual input into data analysis. DF conceived the study, participated in its design and drafted the manuscript. All authors read and approved the final manuscript.

## Supplementary Material

Additional file 1: Table S1 Alignment of tags derived from mouse atria with miRNA hairpins as listed in miRBase version 18. Click here for file

Additional file 2: Table S2 Alignment of tags derived from human right atrial appendage with miRNA hairpins as listed in miRBase version 18. Click here for file

Additional file 3: Table S3 Sequences and abundance of different 5^′^ and 3^′^ murine miR-133a isomiRs identified by sequencing of murine atria. Click here for file

Additional file 4: Table S4 Sequences and abundance of different 5^′^ and 3^′^ human miR-133a isomiRs identified by sequencing of human atria. Click here for file

Additional file 5: Table S5 TargetScan outputs for human miR-133a-3p and miR-133a-5p (from Figure 2C) that were used as inputs for Ingenuity Pathway Analysis. Click here for file
