# Supplementary material for: A heterozygous variant in the human cardiac miR-133 gene, MIR133A2, alters miRNA duplex processing and strand abundance
Source: BMC Genet. 2013 Mar 6;14:18. doi: 10.1186/1471-2156-14-18 (PMC3599331; doi:10.1186/1471-2156-14-18)
Supplement: Additional file 1: Table S1 — Alignment of tags derived from mouse atria with miRNA hairpins as listed in miRBase version 18. [file 1471-2156-14-18-S1.html]

Supplementary Table 1: Relative miRNA abundance in mouse atrial tissue (2x adult WT mice)

  
  
  
If this is the only text that loads, enable javascript in your browser.

T
