# Supplementary material for: A heterozygous variant in the human cardiac miR-133 gene, MIR133A2, alters miRNA duplex processing and strand abundance
Source: BMC Genet. 2013 Mar 6;14:18. doi: 10.1186/1471-2156-14-18 (PMC3599331; doi:10.1186/1471-2156-14-18)
Supplement: Additional file 2: Table S2 — Alignment of tags derived from human right atrial appendage with miRNA hairpins as listed in miRBase version 18. [file 1471-2156-14-18-S2.html]

Supplementary Table 2: Relative miRNA abundance in human atrial appendage (2 x adult males)

  
  
  
If this is the only text that loads, enable javascript in your browser.

T
