# Supplementary material for: A heterozygous variant in the human cardiac miR-133 gene, MIR133A2, alters miRNA duplex processing and strand abundance
Source: BMC Genet. 2013 Mar 6;14:18. doi: 10.1186/1471-2156-14-18 (PMC3599331; doi:10.1186/1471-2156-14-18)
Supplement: Additional file 3: Table S3 — Sequences and abundance of different 5′ and 3′ murine miR-133a isomiRs identified by sequencing of murine atria. [file 1471-2156-14-18-S3.pdf]

Supplementary Table 3   mmu-mir-133a-2 aligned reads

```
AGAAGCCAAATGCTTTGCTGAAGCTGGTAAAATGGAACCAAATCAGCTGTTGGATGGATTTGGTCCCCTTCAACCAGCTGTAGCTGCGCATTGATCACGCCGCA    Precursor
                      GCTGGTAAAATGGAACCAAAT               TTTGGTCCCCTTCAACCAGCTG                            5p and 3p mirbase entries

                                                                                                            Length
...........................................................TTGGTCCCCTTCAACCAGCTGT.......................    893,013
..........................................................TTTGGTCCCCTTCAACCAGCTGT.......................    288,924
...........................................................TTGGTCCCCTTCAACCAGCTG........................    34,991
..........................................................TTTGGTCCCCTTCAACCAGCTG........................    32,513
............................................................TGGTCCCCTTCAACCAGCTGT.......................    30,961
...........................................................TTGGTCCCCTTCAACCAG...........................    27,371
...........................................................TTGGTCCCCTTCAACCAGCTGTA......................    26,685
...........................................................TTGGTCCCCTTCAACCAGCTGTAG.....................    17,409
..........................................................TTTGGTCCCCTTCAACCA............................    11,872
..........................................................TTTGGTCCCCTTCAACCAGCTGTA......................    8,958
..........................................................TTTGGTCCCCTTCAACCAG...........................    8,765
...........................................................TTGGTCCCCTTCAACCAGCTGTAGC....................    4,836
..........................................................TTTGGTCCCCTTCAACCAGCT.........................    3,285
...........................................................TTGGTCCCCTTCAACCAGCT.........................    3,025
..........................................................TTTGGTCCCCTTCAACCAGCTGTAG.....................    3,022
............................................................TGGTCCCCTTCAACCAGCTG........................    2,302
..........................................................TTTGGTCCCCTTCAACCAGC..........................    2,076
...........................................................TTGGTCCCCTTCAACCAGC..........................    2,049
............................................................TGGTCCCCTTCAACCAGCTGTA......................    1,793
..........................................................TTTGGTCCCCTTCAACCAGCTGTAGC....................    1,606
............................................................TGGTCCCCTTCAACCAGCTGTAG.....................    1,149
...........................................................TTGGTCCCCTTCAACCAGCTGTAGCT...................    800
.............................................................GGTCCCCTTCAACCAGCTGT.......................    407
----------------------------------------------------------------CCCCTTCAACCAGCTGTA----------------------    285
---------------------------------------------------------------TCCCCTTCAACCAGCTGT-----------------------    280
............................................................TGGTCCCCTTCAACCAGC..........................    275
..........................................................TTTGGTCCCCTTCAACCAGCTGTAGCT...................    260
............................................................TGGTCCCCTTCAACCAGCT.........................    171
--------------------------------------------------------------GTCCCCTTCAACCAGCTGT-----------------------    158
.....................AGCTGGTAAAATGGAACCAAAT.............................................................    155
.....................AGCTGGTAAAATGGAACCAAA..............................................................    144
.........................................................ATTTGGTCCCCTTCAACCAGCTGT.......................    108
-----------------------------------------------------------------CCCTTCAACCAGCTGTAGC--------------------    90
...........................................................TTGGTCCCCTTCAACCAGCTGTAGCTG..................    89
............................................................TGGTCCCCTTCAACCAGCTGTAGC....................    88
.....................AGCTGGTAAAATGGAACCAA...............................................................    51
.....................AGCTGGTAAAATGGAACCAAATC............................................................    49
...........................................................TTGGTCCCCTTCAACCAGCTGTAGCTGC.................    39
.............................................................GGTCCCCTTCAACCAGCTG........................    38
.....................AGCTGGTAAAATGGAACC.................................................................    36
..........................................................TTTGGTCCCCTTCAACCAGCTGTAGCTG..................    36
.............................................................GGTCCCCTTCAACCAGCTGTA......................    36
.............................................................GGTCCCCTTCAACCAGCTGTAG.....................    29
.........................................................ATTTGGTCCCCTTCAACCAGCTG........................    27
--------------------------------------------------------------GTCCCCTTCAACCAGCTG------------------------    25
--------------------------------------------------------------------------------TAGCTGCGCATTGATCACGC----    25
-----------------------------------------------------------------CCCTTCAACCAGCTGTAG---------------------    23
---------------------------------------------------------------TCCCCTTCAACCAGCTGTA----------------------    23
----------------------------------------------------------------CCCCTTCAACCAGCTGTAGC--------------------    22
........................................................GATTTGGTCCCCTTCAACCAGCTGT.......................    18
..........................................................TTTGGTCCCCTTCAACCAGCTGTAGCTGC.................    18
............................................................TGGTCCCCTTCAACCAGCTGTAGCT...................    18
------------------------------------------------------------------CCTTCAACCAGCTGTAGC--------------------    18
....................AAGCTGGTAAAATGGAACCAAA..............................................................    16
.........................................................ATTTGGTCCCCTTCAACC.............................    15
--------------------------------------------------------------GTCCCCTTCAACCAGCTGTA----------------------    15
.....................AGCTGGTAAAATGGAACCA................................................................    14
---------------------------------------------------------------TCCCCTTCAACCAGCTGTAG---------------------    14
--------------------------------------------------------------GTCCCCTTCAACCAGCTGTAG---------------------    12
----------------------------------------------------------------CCCCTTCAACCAGCTGTAG---------------------    12
```

```
......................GCTGGTAAAATGGAACCAAA..............................................................   11
.........................................................ATTTGGTCCCCTTCAACCAGC..........................   12
.........................................................ATTTGGTCCCCTTCAACCAGCT.........................   11
.........................................................ATTTGGTCCCCTTCAACCA............................   10
....................AAGCTGGTAAAATGGAACCAA...............................................................   9
......................GCTGGTAAAATGGAACCAAAT.............................................................   8
......................GCTGGTAAAATGGAACCAAATC............................................................   8
...........................................................TTGGTCCCCTTCAACCAGCTGTAGCTGCGC...............   8
---------------------------------------------------------------------------------AGCTGCGCATTGATCACGC----   8
.........................................................ATTTGGTCCCCTTCAACCAG...........................   7
------------------------------------------------------TGGATTTGGTCCCCTTCAACCAGCTGT-----------------------   6
.........................................................ATTTGGTCCCCTTCAACCAGCTGTA......................   6
..........................................................TTTGGTCCCCTTCAACCAGCTGTAGCTGCGC...............   6
........................................................GATTTGGTCCCCTTCAAC..............................   5
-------------------------------------------------------------------CTTCAACCAGCTGTAGCT-------------------   5
.....................AGCTGGTAAAATGGAACCAAATCA...........................................................   4
.......................................................GGATTTGGTCCCCTTCAACCAGCTGT.......................   4
--------------------------------------------------------------GTCCCCTTCAACCAGCTGTAGC--------------------   4
--------------------------------------------------------------------------------TAGCTGCGCATTGATCAC------   4
............................................................TGGTCCCCTTCAACCAGCTGTAGCTG..................   3
.............................................................GGTCCCCTTCAACCAGCTGTAGC....................   3
....................AAGCTGGTAAAATGGAAC..................................................................   2
....................AAGCTGGTAAAATGGAACCAAAT.............................................................   2
......................GCTGGTAAAATGGAACCAA...............................................................   2
......................GCTGGTAAAATGGAACCAAATCA...........................................................   2
........................................................GATTTGGTCCCCTTCAACCAGCTG........................   2
.........................................................ATTTGGTCCCCTTCAACCAGCTGTAG.....................   2
..........................................................TTTGGTCCCCTTCAACCAGCTGTAGCTGCGCA..............   2
.............................................................GGTCCCCTTCAACCAGCTGTAGCT...................   2
---------------------------------------------------------------TCCCCTTCAACCAGCTGTAGC--------------------   2
-----------------------------------------------------------------CCCTTCAACCAGCTGTAGCT-------------------   2
....................AAGCTGGTAAAATGGAACC.................................................................   1
.......................CTGGTAAAATGGAACCAA...............................................................   1
--------------------------GTAAAATGGAACCAAATCAGCTGTT-----------------------------------------------------   1
------------------------------------------------------TGGATTTGGTCCCCTTCA--------------------------------   1
------------------------------------------------------TGGATTTGGTCCCCTTCAACCAGCTG------------------------   1
........................................................GATTTGGTCCCCTTCAACCAGCTGTAG.....................   1
..........................................................TTTGGTCCCCTTCAACCAGCTGTAGCTGCG................   1
...........................................................TTGGTCCCCTTCAACCAGCTGTAGCTGCGCA..............   1
............................................................TGGTCCCCTTCAACCAGCTGTAGCTGC.................   1
.............................................................GGTCCCCTTCAACCAGCT.........................   1
---------------------------------------------------------------TCCCCTTCAACCAGCTGTAGCT-------------------   1
--------------------------------------------------------------------------------------CGCATTGATCACGCCGCA   1
```
