# Supplementary material for: A heterozygous variant in the human cardiac miR-133 gene, MIR133A2, alters miRNA duplex processing and strand abundance
Source: BMC Genet. 2013 Mar 6;14:18. doi: 10.1186/1471-2156-14-18 (PMC3599331; doi:10.1186/1471-2156-14-18)
Supplement: Additional file 4: Table S4 — Sequences and abundance of different 5′ and 3′ human miR-133a isomiRs identified by sequencing of human atria. [file 1471-2156-14-18-S4.pdf]

Supplementary Table 4 hsa-miR-133a-2 aligned reads

GGGAGCCAAATGCTTTGCTAGAGCTGGTAAAATGGAACCAAATCGACTGTCCAATGGATTTGGTCCCCTTCAACCAGCTGTAGCTGTGCATTGATGGCGCCG	Precursor
TTTGGTCCCCTTCAACCAGCTG	miRBase 3p entry
	Sum counts
....TTGGTCCCCTTCAACCAGCTGT.....................	264,516
....TTTGGTCCCCTTCAACCAGCTGT.....................	192,568
....TTTGGTCCCCTTCAACCAGCTG......................	21,061
....TTGGTCCCCTTCAACCAGCTGTA....................	18,422
....TGGTCCCCTTCAACCAGCTGT.....................	15,132
....TTGGTCCCCTTCAACCAGCTGTAG...................	10,156
....TTGGTCCCCTTCAACCAG.........................	9,365
....TTGGTCCCCTTCAACCAGCTG......................	8,660
....TTTGGTCCCCTTCAACCAGCTGTA....................	7,277
....TTTGGTCCCCTTCAACCAGCT.......................	4,116
....TTTGGTCCCCTTCAACCAG.........................	3,790
....TTTGGTCCCCTTCAACCA..........................	3,109
....TGGTCCCCTTCAACCAGCTGTA....................	1,891
....TTTGGTCCCCTTCAACCAGCTGTAG...................	1,858
....TGGTCCCCTTCAACCAGCTGTAG...................	1,489
....TTGGTCCCCTTCAACCAGCT.......................	1,478
....TTTGGTCCCCTTCAACCAGC........................	1,352
....TTGGTCCCCTTCAACCAGCTGTAGC..................	1,081
....TTGGTCCCCTTCAACCAGC........................	822
....TGGTCCCCTTCAACCAGCTG......................	718
....TTTGGTCCCCTTCAACCAGCTGTAGC..................	408
....TTTGGTCCCCTTCAACCAGCTGTAGCT.................	391
....TTGGTCCCCTTCAACCAGCTGTAGCT.................	262
---------------------AGCTGGTAAAATGGAACCAAA--	198
---------------------AGCTGGTAAAATGGAACCAAAT---	195
....GGTCCCCTTCAACCAGCTGT.....................	180
....TGGTCCCCTTCAACCAGC........................	152
---TCCCCTTCAACCAGCTGT---------------------	132
--CCCCTTCAACCAGCTGTA--------------------	119
....TGGTCCCCTTCAACCAGCTGTAGC..................	90
--GTCCCCTTCAACCAGCTGT---------------------	89
....TGGTCCCCTTCAACCAGCT.......................	64
---------------------AGCTGGTAAAATGGAACCAA---	57
---CCCTTCAACCAGCTGTAGC------------------	52
....ATTTGGTCCCCTTCAACCAGCTGT.....................	49
---------------------AGCTGGTAAAATGGAACCAAATC--	48
---TCCCCTTCAACCAGCTGTA--------------------	30
--CCTTCAACCAGCTGTAGC------------------	28
---------------------AGCTGGTAAAATGGAACC---	25
....GGTCCCCTTCAACCAGCTGTAG...................	24
....GGTCCCCTTCAACCAGCTGTA....................	18
---------------------AGCTGGTAAAATGGAACCA--	16
....GGTCCCCTTCAACCAGCTG......................	16
....TTGGTCCCCTTCAACCAGCTGTAGCTG................	15
---CCCTTCAACCAGCTGTAG-------------------	15
----------------------GCTGGTAAAATGGAACCAAAT---	14
----------------------GCTGGTAAAATGGAACCAAATC--	12
....TTGGTCCCCTTCAACCAGCTGTAGCTGT...............	11
....ATTTGGTCCCCTTCAACCAGCTG......................	10
....TTTGGTCCCCTTCAACCAGCTGTAGCTGTG..............	10
--------------------GAGCTGGTAAAATGGAACCAAA--	9
....TTTGGTCCCCTTCAACCAGCTGTAGCTGT...............	9
....TTTGGTCCCCTTCAACCAGCTGTAGCTGTGC.............	9
--GTCCCCTTCAACCAGCTGTA--------------------	8
---TCCCCTTCAACCAGCTGTAG-------------------	8
--CCCCTTCAACCAGCTGTAG-------------------	8
....TTGGTCCCCTTCAACCAGCTGTAGCTGTGC.............	7
....TGGTCCCCTTCAACCAGCTGTAGCT.................	7
--GTCCCCTTCAACCAGCTG----------------------	7
----------------------GCTGGTAAAATGGAACCAAA--	6
....TTGGTCCCCTTCAACCAGCTGTAGCTGTG..............	6
--------------------GAGCTGGTAAAATGGAACCAA---	5
....GATTTGGTCCCCTTCAACCAGCTGT.....................	5

--CCCCTTCAACCAGCTGTAGC------------------ 5

---CTTCAACCAGCTGTAGCT----------------- 5

------------------------TGGTAAAATGGAACCAAATCGA-- 4

--TGGATTTGGTCCCCTTCAACCAGCTGT--------------------- 4

....ATTTGGTCCCCTTCAACCAGCTGTAG................... 3

....TTTGGTCCCCTTCAACCAGCTGTAGCTG................ 3

--GTCCCCTTCAACCAGCTGTAG------------------- 3

....GGTCCCCTTCAACCAGCT....................... 3

------------------------TGGTAAAATGGAACCAAA-- 2

---ATGGATTTGGTCCCCTTCAACCAGCTGT--------------------- 2

....TGGTCCCCTTCAACCAGCTGTAGCTG................ 2

....TGGTCCCCTTCAACCAGCTGTAGCTGT............... 2

....GGTCCCCTTCAACCAGCTGTAGC.................. 2

---TCCCCTTCAACCAGCTGTAGCT----------------- 2

---AGCTGTGCATTGATGGCGC-- 2

--GAGCCAAATGCTTTGCTAG--- 1

--GAGCCAAATGCTTTGCTAGA-- 1

--------------------GAGCTGGTAAAATGGAAC-- 1

--------------------GAGCTGGTAAAATGGAACC--- 1

--------------------GAGCTGGTAAAATGGAACCA-- 1

--------------------GAGCTGGTAAAATGGAACCAAAT--- 1

----------------------GCTGGTAAAATGGAACCAAATCG--- 1

-----------------------CTGGTAAAATGGAACCAA--- 1

------------------------TGGTAAAATGGAACCAAATCGACTGT-- 1

------------------------TGGTAAAATGGAACCAAATCGACTGTCC-- 1

-------------------------------------CCAAATCGACTGTCCAATGGATTT--- 1

--CCAATGGATTTGGTCCCCTTCAACCAGCTG---------------------- 1

---CAATGGATTTGGTCCCCTTCAACCAGCTGT--------------------- 1

....GGATTTGGTCCCCTTCAACCAGCT....................... 1

....GGATTTGGTCCCCTTCAACCAGCTGT..................... 1

....GATTTGGTCCCCTTCAACC........................... 1

....GATTTGGTCCCCTTCAACCAGCTGTA.................... 1

....ATTTGGTCCCCTTCAACCAG......................... 1

....ATTTGGTCCCCTTCAACCAGC........................ 1

....ATTTGGTCCCCTTCAACCAGCT....................... 1

....ATTTGGTCCCCTTCAACCAGCTGTA.................... 1

....TTGGTCCCCTTCAACCAGCTGTAGCTGTGCA............ 1

....TTGGTCCCCTTCAACCAGCTGTAGCTGTGCAT........... 1

....TTGGTCCCCTTCAACCAGCTGTAGCTGTGCATTG......... 1

--GTCCCCTTCAACCAGCTGTAGC------------------ 1

--TAGCTGTGCATTGATGGCGC-- 1

---AGCTGTGCATTGATGGCG--- 1
